# Global mental health: the role of collaboration during the COVID-19 pandemic

**DOI:** 10.1017/gmh.2021.20

**Published:** 2021-05-28

**Authors:** Kimberly Hook, Haley A. Carroll, Elizabeth F. Louis, Maria C. Prom, Amelia M. Stanton, Sergiy Bogdanov, Bonginkosi Chiliza, Luisa Feline Freier, Godfrey Zari Rukundo, Senait Ghebrehiwet, Christina P. C. Borba, Gregory L. Fricchione, David C. Henderson

**Affiliations:** 1 Department of Psychiatry, Boston Medical Center, Boston, MA, USA; 2 Department of Psychiatry, Boston University School of Medicine, Boston, MA, USA; 3 Department of Psychiatry, Massachusetts General Hospital, Boston, MA, USA; 4 National University of Kyiv-Mohyla Academy, Center for Mental Health and Psychosocial Support, Kyiv, Ukraine; 5 Department of Psychiatry, University of KwaZulu-Natal, Nelson R. Mandela School of Medicine, KwaZulu-Natal, South Africa; 6 Department of Social and Political Sciences, Universidad del Pacífico, Lima, Perú; 7 Department of Psychiatry, Mbarara University of Science and Technology, Mbarara, Uganda; 8 Department of Psychiatry, Harvard Medical School, Boston, MA, USA; 9 Harvard T.H. Chan School of Public Health, Boston, MA, USA

Collaboration is the cornerstone of effective global mental health (GMH) practice and fosters international partners’ ability to share global knowledge and local expertise (Godoy-Ruiz *et al*., 2016; Kohrt *et al*., 2016; Hook and Vera, 2020). Developing strong professional relationships, with emphasis on communication, trust, and respect, is vital for impactful collaborations. Substantive professional relationships built through collaboration buffer the negative effects of challenges and maintains morale during difficulties (Khenti *et al*., 2016; Hook and Vera, 2020). Critically, collaboration plays a key role in allowing global partners to manage and persevere during the current time.

There is a strong ethical imperative for international collaborations. Historically, global emergencies have frequently resulted in cultural outsiders, including medical and mental health professionals, responding to disasters in ways that may inadvertently result in more harm than good (Wessells, 2009; Watters, 2010; Shah, 2011). Research is not immune from these issues; other authors have already drawn attention to the possibility that coronavirus disease-2019 (COVID-19) responses may drive exclusionary global health projects (Abimbola *et al*., 2021). Here, collaborations that prioritize the expertise and perspective of local partners offer an opportunity for clinical and research responses that are culturally responsive and grounded in the local context. Developing meaningful, as opposed to token, reciprocal relationships is utmost priority in order to conduct ethical, acceptable, and sustainable research (e.g. Kohrt *et al*., 2014; Grillo *et al*., 2019; Osborn *et al*., 2020).

Fostering strong collaborations will be especially important when considering how global teams may work together to meet the growing mental health needs that are expected after the COVID-19 pandemic (Taquet *et al*., 2021). In addition to addressing needs, the pandemic has highlighted systemic challenges (e.g. economic hardships, strained healthcare systems, digital divides) in both resourced and under-resourced settings (The Lancet, 2021). Increased emphasis and ongoing investment in global partnerships is needed to meet these immediate needs and address large-scale issues over time.

The authors of this commentary believe that strong relationships, and in turn effective collaborations, are key in continuing to conduct GMH research generally and specifically in response to the COVID-19 pandemic. From the perspective of researchers in the United States (US), Peru, South Africa, Uganda, and Ukraine, we discuss the role of collaboration as experienced throughout the ongoing pandemic. Specifically, we highlight two key domains that have been especially relevant over the past months − technology and funding – and discuss their impacts on our global collaborations, considering how these lessons learned may be relevant as we move forward.

## Technology

Engaging with technology facilitates the collaborative relationships that are required to initiate, conduct, and disseminate GMH research. Prior to the start of the pandemic, many international collaborators were already using platforms like Zoom and Skype to conduct regular meetings, discuss any ongoing challenges executing study protocols, and plan future projects. Collaborators were also frequently traveling back and forth between countries for in-person meetings, to identify appropriate study sites, and to plan for study start-up. In the context of the pandemic, these in-person gatherings and study planning visits are no longer possible, creating both opportunities to strengthen partnerships through the use of technology, as well as address challenges that strained collaborative relationships.

Several examples demonstrate how the spread of COVID-19 normalized international collaborations and strengthened existing relationships that already relied on technology. For example, the Africa Global Mental Health Institute (AGMHI) (Grillo *et al*., 2019), which was established in 2016 to reduce the burden of mental illness and build the infrastructure necessary to make systematic changes in mental health care delivery across the African continent, offered a series of lectures delivered via Zoom that discussed strategies for bidirectional capacity-building during the pandemic. Participants based in African countries and in the US shared their experiences managing COVID-related challenges. Given the dynamic nature of the pandemic, some regions experienced high prevalence rates before others, so those with more experience managing the effects of the virus were able to share lessons learned. The pandemic also enabled partners to leverage existing technological resources, such as WhatsApp, to communicate and ensure that projects that could continue did so relatively smoothly. Additionally, due to rapid changes by educational institutions in adopting online learning, global partners were able to support one another by providing Zoom-based lectures and instruction to each other's students worldwide. This was particularly useful when COVID-19 caused physical illness or time pressures among individuals on our respective teams, and the combination of our partnerships and use of novel forms of technology offered a tangible mechanism to be of practical assistance to one another. Finally, using technology in novel ways, such as offering mental health support via telemedicine to healthcare providers, improved intra-country partnerships and suggested future opportunities to provide more readily accessed mental health care.

Though digital resources created opportunities for sustained collaboration, a number of technological challenges also became evident. Even though study teams may have had the tools to work remotely (e.g. laptop computers, mobile phones, WiFi capability), WiFi signals in rural areas of Ukraine, South Africa, and Uganda, where some of our collaborators are based, have complicated work from home efforts. Some team members who were unable to work from home due to poor WiFi connection have decided to look for employment elsewhere, increasing workload on managers or supervisors and exacerbating digital fatigue. As the pandemic has decreased face-to-face interactions, which have strong cultural value in many of the contexts in which we work, there has been some discomfort with new technology. Given established cultural norms, the hesitation of some research participants to interact with newly established research contacts during the course of the pandemic is certainly understandable, though it has contributed to delays in executing projects and hindered collaborative efforts to some degree.

## Funding

Obtaining funding for GMH is an ongoing challenge (Wainberg *et al*., 2017). Gaining and maintaining financial support is a necessary element of GMH and is deeply integrated within collaborative partnerships. The importance of addressing mental health needs was brought to the forefront during the COVID-19 pandemic, which resulted in new funding pathways as the world seeks to understand both the virus and its psychosocial impact. In some of our experiences, these new opportunities resulted in expanding current work and possible new grant submissions. Examples include opportunities to seek additional funding by widening current studies to address the mental health impact of COVID-19 among new target groups (e.g. Peru and Ukraine). For others, COVID-19 grants opened windows for new intra- and inter-country collaborations. A new collaboration developed among researchers in Kenya, Nigeria, South Africa, and Uganda, stemming from knowledge sharing through the AGMHI, is a prime example, whereby partners sought collaborative COVID-19 specific grants aiming to evaluate and address mental health needs among mental health care workers across countries.

Nonetheless, the clinical and administrative burden of COVID-19 illness on clinician-researchers also led to decreased time and energy available to dedicate to research and grant development. For instance, some teams described feeling discouraged: although new funding mechanisms were developed, time to develop applications was constrained due to overwhelming clinical caseloads. Other partners described changes in their collaborations, including loss of team members (e.g. moving out of country, lack of availability of previous partners) that made it nearly impossible to competitively apply for high-level grant mechanisms. In addition to collaboration challenges leading to difficulties with gaining new funding, GMH researchers also face concerns that existing funding will be lost or diverted to COVID-19 related research, which may have negative implications for global collaborations. Loss of collaborators, and in turn potential loss of funding, further challenges existing regional divides and already limited mental health research capacity that is present in many global settings (Razzouk *et al*., 2010; Wainberg *et al*., 2017; Gewin, 2020).

## Looking ahead

The COVID-19 pandemic is a unique time in history that directly impacts the mental health needs of individuals worldwide (Adhanom Ghebreyesus, 2020; Gruber *et al*., 2020). We anticipate that collaborations will be critical in addressing these vulnerabilities, specifically those that focus on offering culturally responsive, innovative interventions to meet growing mental health needs, that bolster international capacity building efforts, and that conduct rigorous research to better understand the mental health impacts from the pandemic.

As described above, we observed over the past months that strong collaborative relationships influence the success and sustainability of mental health research and interventions, particularly when faced with obstacles like those seen during the COVID-19 pandemic. At times, opportunities to strengthen collaborations emerged despite and often because of challenges caused by the pandemic. Many existing partnerships were reinforced by opportunities to demonstrate the reciprocity and ongoing commitment needed to sustain global collaborations. As the intensity of COVID-19 impacted global regions at different time points, opportunities to learn from other's experiences emerged in a tangible way, allowing our partnerships to improve and inform our responses. Nevertheless, we also recognize that barriers inevitably challenged some of our collaborations: time demands resulted in changes in availability, some partners were personally and professionally overburdened, and previous collaborations disintegrated.

At this time, the long-term impacts of COVID-19 on GMH research processes are unknown. We anticipate that worldwide recovery from the pandemic will be longstanding; for example, it is already clear that inequities in accessing vaccines will impact our global partners over several years (Aryeetey *et al*., 2021). In light of collaboration's key role in maintaining resilience and ensuring cultural relevance, we call for the GMH community to recommit to the importance of long-standing, effective international partnerships. Maintaining collaborations during COVID has allowed our international teams: to better understand and respond to community needs and priorities; to consider new opportunities and pathways for future work; to build trust and understanding that our partnerships are enduring despite grave difficulties; and to learn to be flexible in responding to rapidly changing environments. When subsequent challenges inevitably impact our partnerships, we believe that some of the lessons learned in navigating the pandemic may serve as a blueprint for future efforts. We emphasize the importance of bidirectional knowledge sharing that can strengthen our ability to recover and grow in a future post-pandemic world (White *et al*., 2014). It is our hope and belief that the lessons learned from the compounded circumstances and the strengths that are emerging will guide and bolster collaborative GMH partnerships, both currently and in future efforts.
